# The incidence of ventilator-associated pneumonia using the PneuX System with or without elective endotracheal tube exchange: A pilot study

**DOI:** 10.1186/1756-0500-4-92

**Published:** 2011-03-30

**Authors:** Alex Doyle, Andrew Fletcher, Joseph Carter, Mark Blunt, Peter Young

**Affiliations:** 1 Department of Anaesthesia and Critical Care, Queen Elizabeth Hospital, Kings Lynn, Norfolk, PE30 4ET, UK

## Abstract

**Background:**

The PneuX System is a novel endotracheal tube and tracheal seal monitor, which has been designed to minimise the aspiration of oropharyngeal secretions. We aimed to determine the incidence of ventilator-associated pneumonia (VAP) in patients who were intubated with the PneuX System and to establish whether intermittent subglottic secretion drainage could be performed reliably and safely using the PneuX System.

**Findings:**

In this retrospective observational study, data was collected from 53 sequential patients. Nine (17%) patients were initially intubated with the PneuX System and 44 (83%) patients underwent elective exchange to the PneuX System. There were no episodes of VAP while the PneuX System was *in situ*. On an intention to treat basis, the incidence VAP was 1.8%. There were no complications from, or failure of, subglottic secretion drainage during the study.

**Conclusions:**

Our study demonstrates that a low incidence of VAP is possible using the PneuX System. Our study also demonstrates that elective exchange and intermittent subglottic secretion drainage can be performed reliably and safely using the PneuX System.

## Introduction

Ventilator-associated pneumonia (VAP) can be defined as a pneumonia that occurs after more than 48 hours of intubation and mechanical ventilation [1]. The incidence and mortality of VAP has been estimated to lie between 10-20% and 15-50% respectively, and it has been shown to extend the length of stay in the intensive care unit (ICU) and costs by at least 6 days and $10019 per episode [2]. With these figures in mind, the prevention of VAP is of paramount importance. The aspiration of contaminated secretions from the oropharyngeal space is by far the most common cause of VAP [3]. Risk factors for the aspiration of oropharyngeal secretions in the critically ill include [4]:

• Reintubation

• Accumulation of secretions above the endotracheal tube cuff

• Colonisation of the upper airway with nosocomial pathogens

• Inappropriate endotracheal tube cuff pressure

• Bacterial colonisation and biofilm formation inside the endotracheal tube lumen

Subglottic secretion drainage (SSD) has been recommended to minimise the accumulation of secretions above the endotracheal tube (ETT) cuff [4-6]. This has been shown to reduce the incidence of VAP by approximately 50%, but requires the use of a compatible device such as the Hi-Lo Evac, SealGuard Evac, TaperGuard (Covidien, Massachusetts, USA), Portex SACETT (Portex, Hythe, UK) and the Teleflex ISIS HVT (Teleflex, Wisconsin, USA) [4,7,8]. Frequently, patients are intubated with a conventional ETT in the first instance. It should be noted that there is no evidence to suggest that electively reintubating a patient to exchange a conventional ETT to a SSD compatible device is associated with VAP [9].

**Figure 1:**
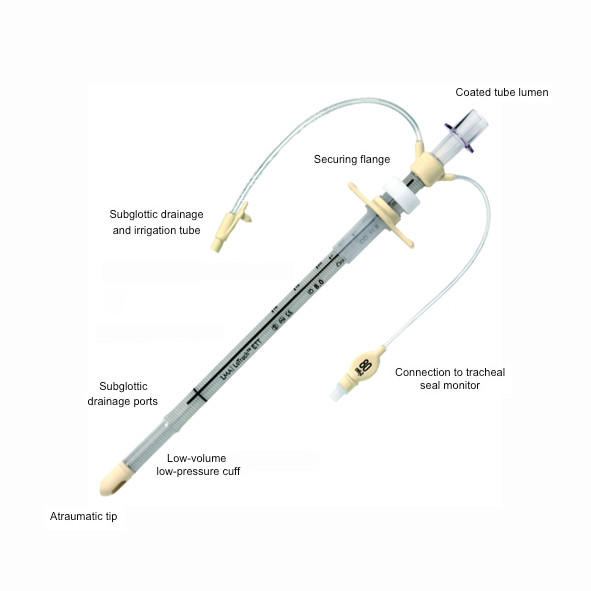
**The PneuX System endotracheal tube**. Cuff is shown deflated. The subglottic drainage ports lie immediately superior to the cuff.

**Figure 2:**
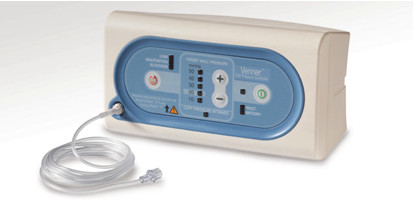
**The PneuX System tracheal seal monitor**.

The PneuX System (Venner Medical, Singapore) comprises of a novel ETT and tracheal seal monitor (figure 1 and 2). Unlike the other SSD compatible devices, the PneuX System incorporates several strategies to minimise the aspiration of oropharyngeal secretions [10]. These include a securing flange, a unique low-volume low-pressure (LVLP) cuff, multiple SSD ports, a tracheal seal monitor and a coated tube lumen.

We aimed to determine the incidence of VAP in patients who were initially intubated with the PneuX System (primary intubation) or those who underwent elective exchange to the PneuX System in our ICU. We also aimed to establish whether intermittent subglottic secretion drainage could be performed reliably and safely using the PneuX System.

## Methods

This retrospective observational study was conducted at the Queen Elizabeth Hospital, Norfolk, UK with approval from the Local Research and Ethics Committee. Prospective approval was obtained to report data from our routine practice and for publication of anonymised data. Written informed consent was received for publication of the figures. All equipment was used according to the manufacturers' instructions.

Each patient was more than 18 years of age and required intubation as part of his or her routine respiratory care. The use of the PneuX System was restricted to those patients who were anticipated to require more than 24 hours of intubation. All patients included in the study were intubated with the PneuX System and received mechanical ventilation for more than 48 hours. No patients intubated with the PneuX System were excluded from our analysis.

For the purpose of this study, VAP was defined as any pneumonia that occurred after more than 48 hours of intubation and mechanical ventilation with the PneuX System. Ventilator-associated pneumonia was diagnosed by (i) clinical suspicion (including the use of any antibiotics for the treatment of colonisation or infection within the tracheobronchial tree or lungs); and/or (ii) international consensus criteria: the presence of new, persistent pulmonary infiltrates not otherwise explained, appearing on chest radiographs and at least two of the following criteria: temperature of > 38°C; leukocytosis > 10,000 cells/mm^3 ^and purulent respiratory secretions [11]; and/or (iii) clinical pulmonary infection score (a fall in the PaO_2_/FiO_2 _ratio >25% and a clinical pulmonary infection score of >5 in the presence of bacteria in a qualitative endotracheal aspirate).

Elective exchange was defined as elective reintubation to change a conventional ETT to the PneuX System. If a patient had previously been intubated with a conventional ETT, elective exchange was performed once the attending clinician predicted that at least a further 24 hours of ventilation was required. The procedure involved using gastric tube aspiration, pre-procedural pre-oxygenation, muscle relaxation and sedation, direct laryngoscopy to clear upper airway secretions and a bougie (appendix 1).

A standard protocol for the prevention of VAP was used during the study period. This required patients to be nursed in the head up position (between 30-45 degrees) and intermittent SSD to be performed at 4 hourly intervals. Decontamination of the subglottic space, larynx, pharyngeal space and oral cavity was performed using supracuff irrigation with a large volume of normal saline (appendix 2). The cuff pressure was maintained for the duration that the PneuX System was *in situ *by the tracheal seal monitor alone. Airway humidification was provided using a RT dual heated wire circuit, with MR290 Autofeed Humidification Chamber (Fisher and Paykel, Auckland, New Zealand). No new interventions for the prevention of VAP were initiated during the study period other than the use of the PneuX System. Neither oral chlorhexidine nor oral antibiotic pastes were administered.

The data collected included patient age, gender, acute physiology and chronic health evaluation II score, reason for ICU admission, whether the patient was intubated prior to ICU admission, whether a primary intubation or elective exchange was performed, whether there was any complication associated with elective exchange, duration of intubation, the incidence of VAP, all microbiological data in addition to surveillance qualitative endotracheal aspirates, which were performed 2-3 times per week, and the mortality rate. The prescription of antibiotics and reason for treatment was also recorded. The number of SSD interventions and failure of SSD due to blockage of the subglottic ports were recorded. Failure of SSD was defined as an inability to perform SSD because of subglottic drainage port blockage. Data were entered into a spreadsheet and analysed using Excel (Microsoft, California, USA).

## Results

Data was collected from 53 sequential patients. The demographic details are shown in Table 1.

**Table 1:** The patient demographic data.

Variable	Value (SD)
Mean age (years)	67.8 (15)

Gender (female/male)	22/31

Mean APACHE II score	22.4 (8.4)

Intubated prior to admission	21

Reason for ICU admission:	
Cardiovascular	5
Respiratory	24
Gastrointestinal	17
Hepatorenal	2
Metabolic	2
Other	3

Mortality rate in ICU	19

APACHE II score; Acute Physiology and Chronic Health Evaluation II score. ICU; Intensive care unit.

Nine (17%) patients were initially intubated with the PneuX System and 44 (83%) patients underwent elective exchange to the PneuX System. There were no complications associated with elective exchange. There were a total of 306 days of intubation. The mean duration of intubation was 5.3 days +/- 4.1 (median duration of intubation was 5 days and the interquartile range was 2-7 days). There were no episodes of VAP while the PneuX System was *in situ*. On an intention to treat basis, the incidence of VAP was 1.8%: one patient, who had initially been intubated using the PneuX System, underwent planned extubation and required emergency reintubation for respiratory failure. A conventional ETT was used for reintubation and the patient subsequently developed a VAP after 48 hours. Forty eight (91%) patients were treated with antibiotics prior to intubation. Eighteen patients had their antibiotic treatment changed following intubation (14; treatment of the underlying condition, 3; suspected catheter related bloodstream infection and 1; pseudomembranous colitis). No antibiotics were used to treat colonisation or new infection within the tracheobronchial tree or lungs in a patients intubated with the PneuX System. During this time, there were a total of 728 SSD interventions. There was no failure of SSD in a patients intubated with the PneuX System.

## Discussion

Our study demonstrated that there was a low incidence of VAP in patients intubated with the PneuX System, there were no complications associated elective exchange to the PneuX System and intermittent SSD and decontamination of the subglottic space, the larynx, the pharyngeal space and the oral cavity can be performed reliably and safely using the PneuX System.

Previously, the incidence of VAP has been estimated to be much higher in general intensive care patients [2]. The low incidence of VAP in this study may be explained by the fact that the PneuX System minimises a patient's exposure to multiple risk factors for VAP [10]. The relative contribution of each strategy has not been quantified. Therefore, we do not attribute the low incidence of VAP to one strategy in particular.

This is the first article to demonstrate that elective reintubation can be performed safety in the critically ill. However, it should be noted that reintubation *per se *has not been shown to increase the incidence of VAP. The link between reintubation and VAP has only been demonstrated following premature extubation, which may be an accidental extubation or an intentional planned extubation with the subsequent failure of ventilation [9]. Premature extubation leaves the lungs unprotected against pulmonary aspiration. In this setting, premature extubation has been estimated to increase the relative risk of VAP by 5.3 times [12]. Our results suggest that it may be safe to electively exchange a conventional ETT to the PneuX System. This may not be applicable to all other devices. The PneuX System is a flexible armoured tube and has an atraumatic boat-tip [10]. These features have been shown to ease reintubation over an introducer and passage through the glottis when compared to a conventional ETT, which is more rigid and has a bevel tip [13].

Our study also demonstrated that intermittent SSD can be performed reliably and safely using the PneuX System. Other SSD compatible devices have a single subglottic drainage port have been shown to fail on 48% of occasions, most commonly because of blockage of the subglottic drainage port by suctioned tracheal mucosa [14]. The PneuX System has three circumferential subglottic drainage ports, which allow drainage to proceed through the two unoccluded ports should one of them become obstructed by suctioned tracheal mucosa [10].

Other SSD compatible devices also have conventional high volume low pressure cuffs, which have been shown to allow subglottic secretions to leak around the cuff [15]. Consequently, continuous aspiration has been used to prevent the accumulation of secretions above the cuff. However, this may produce ischaemic injury to suctioned tracheal mucosa and therefore intermittent drainage is commonly used [16,17]. In contrast, the PneuX System has a unique LVLP cuff, which has been shown to completely prevent pulmonary aspiration [10,15]. Consequently, it is safe to allow subglottic secretions to build up above the cuff and perform intermittent SSD.

The superior performance of the LVLP cuff also enables decontamination of the subglottic space, the larynx, the pharyngeal space and the oral cavity to be performed using supracuff irrigation with a large volume of normal saline. It was notable that once subglottic drainage had been performed to dryness, a residual offensive collection of upper airway fluid (estimated at 10-30 mL) remained in the laryngopharynx. This residual material was only cleared by irrigation with large volumes of normal saline (up to 300 mL).

An important limitation of this study was that the incidence of VAP before introducing the PneuX System was unknown. Therefore, the impact of introducing the PneuX System could not be quantified. The retrospective nature of our study and the small number of patients included in our analysis from a single institution also represent weaknesses of our study. However, these represented all of the available patients at the time. In light of this exciting pilot data, we now hope to confirm our findings in prospective, multicentre studies with larger patient groups and in independent centres.

In conclusion, our study demonstrates that a low incidence of VAP is possible using the PneuX System. Our study also demonstrates that elective exchange and intermittent subglottic secretion drainage can be performed reliably and safely using the PneuX System.

## List of abbreviations

(VAP): Ventilator-associated pneumonia; (ICU): Intensive care unit. (SSD): Subglottic secretion drainage; (ETT): Endotracheal tube; (LVLP): Low volume low pressure

## Competing interests

Dr Peter Young is the inventor of the PneuX System. In the past he has received funding and consultancy fees from Venner Medical. This work was not funded or financed by Venner Medical. Dr Peter Young is a minor shareholder in the intellectual property ownership of the PneuX System and tracheal seal monitor.

The remaining authors do not declare any conflict of interest or competing interest.

## Authors' contributions

AD interpreted data, drafted the write up and is the lead author of the final version of the manuscript. AF independently acquired and interpreted data, drafted the write up and gave approval for publication. JC independently acquired and interpreted data, made contributions to conception and design, critically revised the manuscript and gave approval for the final version to be published. MB independently acquired and interpreted data, made contributions to conception and design, critically revised the manuscript and gave approval for the final version to be published. PY made contributions to conception and design, interpreted data, critically revised the manuscript and gave approval for the final version to be published.

## Appendix 1. Technique for elective endotracheal tube exchange

1. Empty the stomach using a nasogastric or orogastric tube

2. Apply 100% oxygen for 5 minutes

3. Provide neuromuscular blockade and sedation (as per clinician's judgment)

4. Perform direct laryngoscopy to clear upper airway secretions

5. Use bougie (or other airway devices will be allowed as per clinician's discretion)

6. Once the endotracheal tube is inserted, the cuff will be inflated in all groups with a syringe to an end point of auditory air-seal

7. The endotracheal tube will then be attached to the tracheal seal monitor to achieve air-seal (normally set at 20 mmHg or increased to 30 mmHg to achieve air-seal)

## Appendix 2. The procedure for supracuff irrigation and decontamination of the subglottic space, the larynx, the pharyngeal space and the oral cavity

Aim:

• To irrigate the subglottic space, the larynx, the pharyngeal space and the oral cavity using with 0.9% sodium chloride until visually clean

Frequency:

• Perform once daily or more frequently if either the subglottic aspirates or the oral cavity are offensive

Equipment:

• 50 mL 0.9% sodium chloride at room temperature

• 50 mL Luer syringe

• 10 mL Luer syringe

• Suction unit with Yankeur suction attachment

• Towel to protect the patients neck/chest/bed linen from spillage

• Consider 5 mL 1-2% lignocaine if patient awake or airway irritable

Procedure:

• Use clean technique

• Explain the procedure to the patient if awake

• Tracheal seal monitor set to at least 20 mmHg

• Flush the subglottic port with 5 mL of air to ensure patency

• If patient awake consider pre-instillation of 5 mL lignocaine through port and into subglottic space

• Fill the 50 mL Luer syringe with 50 mL 0.9% sodium chloride

• Attach the syringe to the subglottic port on the Lotrach

• Slowly instill the 50 mL 0.9% sodium chloride over 3-5 minutes whilst the other staff member uses the Yankeur to remove the accumulating fluid from the oral cavity

• If the injection pressure is judged to be high then the tracheal seal monitor will be increased to 50 mmHg for the duration of the irrigation

• 100-300 mL saline is used at the nurse's discretion. The volume used will be determined by the discontinuation of irrigation once the secretions from the mouth become visually non-offensive and predominantly saline

• Once all the saline has been infused, remove any remaining fluid from the subglottic space by attaching the 10 mL Luer syringe to the subglottic port and apply suction over a 10-20 second period or until the flow of fluid ceases

• Finally, remove the giving set from the subglottic port

• During this procedure note any coughing, change in cardiac rhythm and oxygen saturation, and reassure the patient throughout.

• Discontinue if coughing or vagal bradycardia occurs and consider warmer fluid and/or lignocaine
